# Thyroid stimulating hormone, independent of thyroid hormone, can elevate the serum total cholesterol level in patients with coronary heart disease: a cross-sectional design

**DOI:** 10.1186/1743-7075-9-44

**Published:** 2012-05-23

**Authors:** Chao Xu, Xiaomei Yang, Wenhui Liu, Haitao Yuan, Chunxiao Yu, Ling Gao, Jiajun Zhao

**Affiliations:** 1 Department of Endocrinology, Provincial Hospital affiliated to Shandong University, Jinan, China; 2 Institute of Epidemiology and Health Statistics, School of public health, Shandong University, Jinan, China; 3 Cardiovascular Center, Provincial Hospital affiliated to Shandong University, Jinan, China; 4 Institute of Endocrinology, Shandong Academy of Clinical Medicine, Jinan, China; 5 Scientific Center, Provincial Hospital affiliated to Shandong University, Jinan, China

**Keywords:** TSH, Cholesterol, Coronary heart disease

## Abstract

**Background:**

The relationship between TSH and the lipid profile is contradictory because few studies have excluded the potential influence of the thyroid hormones (TH). The aim of the present study was to evaluate the relationship between serum TSH levels and the lipid profile independent of TH.

**Methods:**

1302 CHD patients diagnosed by coronary angiography were retrospectively studied. The prevalence and distribution of thyroid dysfunction were analyzed first. To assess the impact of TSH on serum lipids, Pearson’s correlation analysis was performed after adjustments for classic factors and TH. To calculate the extent of the effect of TSH on the serum cholesterol level, the partial least squares method and additional statistical methods were used.

**Results:**

After the exclusions, a total of 568 patients (270 males and 298 females with a mean age of 63.56 ± 11.376 years) were selected. The prevalence of thyroid dysfunction among the patients was 18.66%, and the prevalence of hypothyroidism (15.32%) was higher than that of hyperthyroidism (3.34%). Even after adjusting for confounding factors, such as sex, age, smoking status, fasting plasma glucose levels and TH, a significant positive impact of TSH on the serum total cholesterol (TC) level was revealed (r = 0.095, *p* = 0.036). Each 1 mIU/L increase in the TSH level might be linked to a 0.015580712 mmol/L elevation of the serum TC value.

**Conclusions:**

TSH can increase the TC level in CHD patients independent of TH. The present study suggests a potential physiological role of TSH and the importance of maintaining an appropriate TSH level in CHD patients.

## Background

The thyroid hormones exert a wide range of functions in several organs, including the heart [1]. Abnormal thyroid hormone metabolism may lead to different forms of heart disease and hypothyroidism, in particular, is a well-known cause of accelerated coronary atherosclerosis [2,3]. Moreover, similar consequences were found for subclinical hypothyroidism (SCH), which is characterized by elevated serum thyroid stimulating hormone (TSH) levels and normal thyroxine (T4) levels [4]. Elevated TSH levels have recently aroused interest due to the potential for TSH to induce injury, especially in patients with coronary heart disease (CHD). A series of studies reported that a high level of TSH was associated with a deleterious change of serum lipids, with an increase of lipid abnormalities [5-8]; however, this issue has been the subject of considerable debate [9], and several studies have not observed such an association [10,11]. The differences in the studies have been ascribed to the influence of some confounding factors, such as age, gender and body mass index (BMI). Existing evidence has demonstrated that the relationship of TSH and lipid levels was different between overweight and normal weight populations and between men and women [12]. Furthermore, the thyroid hormones play an important role in regulating lipid metabolism. Numerous studies have confirmed the presence of an inverse relationship between serum thyroxin and cholesterol levels [2]. Even within the reference range, serum free thyroxine (FT4) levels near the upper limit have been associated with different metabolic markers in euthyroid subjects and patients with coronary artery disease [13,14]. Therefore, to evaluate the essential relationship between TSH and the lipid status, it is necessary to adjust for age, gender, BMI and thyroid hormone levels. Regrettably, few studies have excluded the potential influences of the thyroid hormones when assessing the relationship between TSH and the lipid status.

Interestingly, *in vivo* and *in vitro* research by our laboratory on the function of TSH has shown that TSH, independent of thyroid hormones, can upregulate the expression of hepatic 3-hydroxy-3-methyl-glutaryl coenzyme A reductase (HMGCR), which is the rate-limiting enzyme in cholesterol synthesis, and increase the cholesterol content in the liver [15]. Therefore, we hypothesized that TSH, independent of thyroid hormones, would be positively associated with the serum cholesterol level.

The present study evaluated the relationship between TSH and the lipid status after adjusting for classic confounding factors and the thyroid hormones. We also analyzed the extent to which TSH can affect serum lipid parameters. The present study yielded insights into potentially novel effects of TSH on serum lipids and suggested that it is necessary to routinely test thyroid function in CHD patients. Maintaining serum TSH levels in an appropriate range will achieve homeostasis of the lipid levels and slow the progression of atherosclerosis in CHD patients.

## Materials and methods

### Patients

A total of 1302 patients who were hospitalized in either the Provincial Hospital or the Qianfushan Hospital, which are affiliated with Shandong University (Jinan, China), from 2004 to 2010 were retrospectively reviewed. All of the patients were diagnosed with CHD by coronary angiography according to the international criteria. Information on medication and a history of previous medical or surgical diseases for each patient was obtained. The smoking histories of the patients were also recorded. The blood pressure values were obtained from the medical records and presented as the mean of two measures taken in the sitting position according to a standardized protocol.

The following criteria were used for exclusion: (1) Euthyroid sick syndrome, being characterized by low serum triiodothyronine (T3); (2) Acute myocardial infarction at the moment of hospitalization; (3) Reduced (<50%) left ventricular ejection fraction at echocardiography; (4) Hypothalamus and/or pituitary gland diseases, diabetes mellitus or other endocrine diseases; (5) Intake of drugs that influence serum lipids or thyroid function within the past 3 months; (6) cerebral vascular disease, a malignant tumor, hereditary hyperlipidemia, or serious liver or renal dysfunctions; (7) History of myocardial infarction or revascularization prior to hospitalization; and (8) pregnancy. Generally, the patients were clinically stable at the moment of hospitalization and those with serious condition or in intensive care unit were excluded. In the end, 568 patients (270 males and 298 females with a mean age of 63.56 ± 11.376 years) were selected and enrolled in the present study.

The local ethics committee approved the retrospective review of the patients’ medical records and licensed the records for research purposes only.

### Laboratory analysis

All of the measurements were performed in the clinical laboratory that is affiliated with Shandong University. Blood samples were collected from all of the patients between 8:00 A.M. and 10:00 A.M. after a minimum of a 10-h fast. Chemiluminescent procedures (Cobas E610; Roche, Basel, Switzerland) were employed to determine the thyroid function of the patients, TSH, free triiodothyronine (FT3), FT4 and reverse T3 (rT3). The laboratory reference ranges were 0.27-4.2 mIU/L for TSH, 3.1-6.8 pmol/L for FT3, 12–22 pmol/L for FT4 and 0.54-1.46 nmol/L for rT3. Thyroid function of the patients was measured twice, before hospitalization and the second day after hospitalization. The patients were excluded when distinct variations between the two results were found.

The levels of plasma glucose, total cholesterol (TC), triglycerides (TG), low-density lipoprotein (LDL) cholesterol, high-density lipoprotein (HDL) cholesterol and uric acid (UA) were determined using an Auto Biochemical Analyzer (MODULAR-000GS; Roche, Basel, Switzerland). Hypercholesterolemia was defined as a TC value over 6.21 mmol/L, which is in accordance with the National Cholesterol Education Program Adult Treatment Panel III criteria (NCEP/ATPIII) [16].

All of the CHD patients were initially divided into two groups based on their thyroid function: euthyroid and thyroid dysfunction. Euthyroidism was defined by circulating levels of FT3, FT4 and TSH that were within the reference ranges. The patients with thyroid dysfunction were further divided into the following 3 subgroups: (1) hyperthyroidism, which was classified as a TSH level less than 0.27 mIU/L and/or FT3 and FT4 levels above the reference ranges; (2) subclinical hypothyroidism, which was classified as a TSH level above 4.2 mIU/L and FT3 and FT4 levels in the reference ranges; and (3) hypothyroidism, which was classified as FT3 and FT4 levels less than the reference ranges with a TSH level above 4.2 mIU/L.

### Statistical analyses

Statistical tests were performed in a blinded fashion by two statisticians using SPSS version 17.0 for Windows (Chicago, IL, USA). Parametric and nonparametric data are given as the mean ± SD and the percentage. Groups were compared using a one-way analysis of variance (ANOVA) or Chi-squared test. Variables with a skewed distribution were transformed to their natural logarithm to optimize the models. The relationships between thyroid function and serum lipid parameters were evaluated with Pearson’s correlation analysis. To adjust for the thyroid hormones and several risk factors, a partial correlation analysis and a factorial analysis were performed while evaluating the relationship between TSH and TC. To examine the relationship between TSH and TC, we used four different statistical methods, each with its own advantages. We used linear regression analysis, principal component analysis, the partial least squares method and path analysis. These analyses are often used to analyze similar issues and using them together can compensate for the weaknesses of the individual methods. All of the calculated p-values are two-sided, and *p*-values less than 0.05 are considered statistically significant.

## Results

### Characteristics of the 568 CHD patients according to serum TSH concentrations

The composition and general characteristics of the patients are summarized in Table 1. The normal TSH levels were stratified into three groups (0.27–1.57 mIU/L, 1.58–2.88 mIU/L and 2.89–4.19 mIU/L), and the patients with CHD were divided into 5 groups according to the serum TSH concentrations. Significant differences were detected among the five groups with respect to the levels of FPG (*p* = 0.001), TC (*p* = 0.013), TG (*p* = 0.003), FT3 (*p* < 0.001) and FT4 (*p* < 0.001). No significant differences were found among the five groups with regard to age, SBP, DBP, LDL, HDL or UA.

**Table 1:** The characteristics of 568 CHD patients who were stratified according to serum TSH concentration

		**Serum TSH concentration (mIU/L)**					***P* value**	**Total**
		**<0.27**	**0.27-1.57**	**1.58-2.88**	**2.89-4.19**	**≥4.2**		
Age (y)		64.84(11.64)	65.47(11.27)	61.92(11.54)	63.69(10.57)	64.46(10.82)	0.052	63.76(11.28)
SBP (mm Hg)		137.47(17.01)	134.77(22.72)	132.87(19.45)	136.51(21.17)	136.94(20.35)	0.481	134.74(20.79)
DBP (mm Hg)		80.42(7.77)	79.02(12.54)	79.22(11.96)	78.51(11.83)	80.43(10.54)	0.847	79.27(11.78)
FPG (mmol/L)		6.1328(1.24)	5.4976(1.04)	5.3802(1.30)	5.0863(0.55)	5.2059(0.83)	0.001	5.3733(1.093)
TC (mmol/L)		4.6163 (1.3305)	4.8699(1.03)	4.9598(1.15)	4.9685(1.03)	5.3518(1.34)	0.013	4.9829(1.15)
TG (mmol/L)		1.3226(0.69)	1.3669(0.82)	1.7644(1.62)	1.6951(0.85)	1.9766(1.66)	0.003	1.6538(1.32)
LDL (mmol/L)		3.00(0.851)	2.91(0.77)	2.88(0.841)	2.91 (0.74)	3.16 (0.91)	0.111	2.94(0.82)
HDL (mmol/L)		1.1506(0.29)	1.2891(0.35)	1.3229(0.37)	1.2762(0.32)	1.3557(0.29)	0.141	1.3060(0.34)
UA (μmol/L)		297.9688 (107.94)	313.9742(90.85)	321.0955(101.73)	311.7162(70.90)	321.63(109.47)	0.817	317.0161(95.98)
TSH (mIU/L)		0.1498(0.11)	1.0532(0.34)	2.1371(0.35)	3.5424(0.56)	19.760(33.33)	0.000	4.6504(14.52)
FT3 (pmol/L)		8.1885(6.20)	4.5007(0.79)	4.5055(0.71)	4.5020(0.83)	3.5891(1.24)	0.000	4.4864(1.58)
FT4 (pmol/L)		31.2682(17.85)	17.5750(2.50)	17.3971(2.84)	16.7438(2.44)	13.4475(4.76)	0.000	17.2147(5.30)
Smoking status	A	17	121	159	72	80	0.0000	
	B	0	23	26	3	5	0.0008	
	C	2	23	21	6	1	0.0009	
	D	0	2	5	1	1	0.2222	

1. The data are presented as the mean ± standard deviation (SD). The SD is shown in parentheses.

2. The groups were compared using a Chi-squared test. *P*-values less than 0.05 were considered statistically significant. 3. Abbreviations: SBP, systolic blood pressure; DBP, diastolic blood pressure; FPG, free plasma glucose; TC, total cholesterol; TG, triglycerides; LDL, low-density lipoprotein cholesterol; HDL, high-density lipoprotein cholesterol; and UA, uric acid. 4. The following reference ranges were based on our laboratory standards: Plasma glucose (3.9-6.1 mmol/L), total cholesterol (3.6-6.2 mmol/L), triglycerides (<1.71 mmol/L), low-density lipoprotein cholesterol (0–3.36 mmol/L), high-density lipoprotein cholesterol (>1.15 mmol/L), TSH (0.27–4.2 mIU/L), FT3 (3.1-6.8 pmol/L), FT4 (12–22 pmol/L) and uric acid (142–339 μmol/L). 4. The smoking status categories are as follows: A, B, C and D represent no smoking, smoking 1–339, 440–1099, and above 1100 cigarettes per year, respectively. The data are presented as the number of people in each category.

### The prevalence and distribution of thyroid dysfunction in the CHD patients

Among all of the CHD patients, the prevalence of thyroid dysfunction was 18.66% (Table 2). Interestingly, the prevalence of hypothyroidism (15.32%), including overt and subclinical hypothyroidism, was higher than the prevalence of hyperthyroidism (3.34%). The prevalence of thyroid dysfunction in the females (26.17%) was higher than the prevalence in the males (10.37%).

**Table 2:** The prevalence and distribution of thyroid dysfunction in CHD patients

	**Cases**	**Euthyroid**	**Thyroid dysfunction**		
			**Hyperthyroidism**	**Subclinical hypothyroidism**	**Hypothyroidism**
Male	270	242 (89.63%)	4 (1.48%)	19 (7.04%)	5 (1.85%)
Female	298	220 (73.83%)	15 (5.03%)	38 (12.75%)	25 (8.39%)
Total	568	462 (81.34%)	19 (3.34%)	57 (10.04%)	30 (5.28%)
*p* value		<0.001	<0.01	<0.01	<0.05

The data are presented as the number (percentage) of individuals in each category. The groups were compared using a Chi-squared test. *P*-values less than 0.05 were considered statistically significant.

### The prevalence of hypercholesterolemia in the CHD patients increases with the serum TSH levels

**Figure 1:**
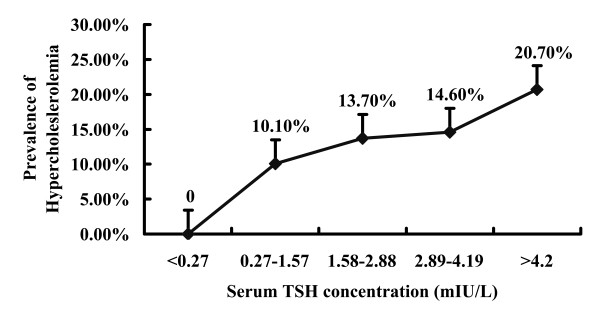
**The prevalence of hypercholesterolemia in CHD patients who were stratified according to the serum TSH concentrations**. The data are presented as the number (percentage) of individuals in each category. The prevalence of hypercholesterolemia showed a linear and significant increase with elevations of the serum TSH levels (Pearson’s Chi-squared test, linear trend 0.010, *p* < 0.05; Figure 1).

In the entire study population, there were 76 cases with hypercholesterolemia, which was approximately 13.5%. The prevalence of hypercholesterolemia showed a linear and significant increase with elevations of the serum TSH levels (Pearson’s Chi-squared test, linear trend 0.010, *p* < 0.05; Figure 1). As expected, the patients with a TSH level below 0.27 mIU/L did not suffer from hypercholesterolemia. However, the prevalence of hypercholesterolemia increased significantly in patients who showed an increased TSH level within the reference range. The prevalence of hypercholesterolemia was also significantly higher in the patients with the serum TSH levels between 2.89 and 4.19 mIU/L compared with the patients with serum TSH levels between 0.27 and 1.57 mIU/L (14.6% vs. 10.1%; *p* < 0.05). The patients with values of TSH greater than or equal to 4.2 mIU/L showed the highest prevalence of hypercholesterolemia (20.7%).

### The relationship between thyroid function and the serum lipid parameters

Because the serum lipid parameters did not fit a normal distribution, log transformations [17] were applied to the values of TC, TG, LDL and HDL (Table 3).

**Table 3:** Correlation analysis of thyroid function and serum lipid parameters in total patients and euthyroidic patients


total patients		LogTC	LogTG	LogLDL	LogHDL
FT3	Unadjusted	r = -0.125	r = -0.010	r = -0.053	r = -0.132
		P = 0.003	P = 0.810	P = 0.212	P = 0.002
	Adjusted*	r = -0.122	r = -0.021	r = -0.057	r = -0.098
		P = 0.007	P = 0.646	P = 0.210	P = 0.030
FT4	Unadjusted	r = -0.190	r = -0.046	r = -0.084	r = -0.184
		P = 0.000	P = 0.269	P = 0.047	P = 0.000
	Adjusted*	r = -0.197	r = -0.035	r = -0.105	r = -0.176
		P = 0.000	P = 0.442	P = 0.021	P = 0.000
TSH	Unadjusted	r = 0.160	r = 0.085	r = 0.047	r = 0.102
		P = 0.000	P = 0.044	P = 0.269	P = 0.015
	Adjusted*	r = 0.146	r = 0.051	r = 0.074	r = 0.069
		P = 0.001	P = 0.261	P = 0.103	P = 0.131
	Adjusted**	r = 0.095	r = 0.044	r = 0.048	r = 0.020
		P = 0.036	P = 0.337	P = 0.295	P = 0.658
euthyroidic patients		LogTC	LogTG	LogLDL	LogHDL
FT3	Unadjusted	r = -0.150	r = -0.113	r = -0.102	r = 0.052
		P = 0.001	P = 0.015	P = 0.029	P = 0.269
	Adjusted*	r = -0.190	r = -0.072	r = -0.141	r = -0.031
		P = 0.000	P = 0.153	P = 0.005	P = 0.537
FT4	Unadjusted	r = -0.012	r = -0.043	r = -0.008	r = 0.140
		P = 0.004	P = 0.355	P = 0.859	P = 0.003
	Adjusted*	r = -0.011	r = -0.057	r = -0.020	r = 0.120
		P = 0.003	P = 0.255	P = 0.690	P = 0.017
TSH	Unadjusted	r = 0.055	r = 0.199	r = 0.003	r = 0.002
		P = 0.040	P = 0.000	P = 0.955	P = 0.974
	Adjusted*	r = 0.050	r = 0.185	r = 0.012	r = -0.010
		P = 0.018	P = 0.001	P = 0.815	P = 0.847
	Adjusted**	r = 0.049	r = 0.190	r = 0.011	r = -0.019
		P = 0.027	P = 0.007	P = 0.821	P = 0.707

TC, TG, LDL, and HDL were transformed to their natural logarithm to optimize the models. *P*-values less than 0.05 were considered statistically significant.

* Adjusted for sex, age, smoking status and FPG.

** Adjusted for sex, age, smoking status, FPG and thyroid hormones.

In total patients, significant negative correlations were identified between the levels of the thyroid hormones (FT3 and FT4) and the serum TC or HDL levels (r = −0.125, *p* = 0.003 for FT3 and LogTC; r = −0.132, *p* = 0.002 for FT3 and LogHDL; r = −0.190, *p* < 0.001 for FT4 and LogTC; and r = −0.184, *p* < 0.001 for FT4 and LogHDL). The levels of FT4 were also negatively correlated with the serum LDL levels (r = −0.084, *p* = 0.047). Interestingly, the correlations remained significant after we adjusted for age, gender, smoking status and FPG. There was no significant relationship between the thyroid hormones (FT3 and FT4) and the serum TG level.

We observed a significant positive impact of TSH on the serum TC, TG and HDL levels (r = 0.160, *p* < 0.001 for TSH and LogTC; r = 0.085, *p* = 0.044 for TSH and LogTG; and r = 0.102, *p* = 0.015 for TSH and LogHDL). Interestingly, the correlations remained significant for the TC level after we adjusted for age, gender, smoking status and FPG (r = 0.146, *p* = 0.001). After an additional adjustment for the thyroid hormone levels (FT3 and FT4), the significance still remained for the TC level (r = 0.095, *p* = 0.036 for TSH and LogTC) but not for the levels of TG, LDL or HDL.

We also conducted statistics in euthyroidic patients separately and the results were shown in Table 3. In general, the results were similar to those conducted in total patients.

### The response relationship between TSH and TC

All of the four different statistical methods revealed that TSH could increase the serum TC level to a certain extent (Data are available upon request). Interestingly, the partial least squares method had advantage on overcoming the adverse effects caused by correlation between the independent variables. Table 4 shows the results of this method. The partial least squares method revealed that each 1 mIU/L increase in TSH was estimated to elevate the TC level by 0.015580712 mmol/L (*p* < 0.001). However, the thyroid hormones (FT3 and FT4) could negatively affect the serum TC level (coefficients = −0.001921199 and −0.011725050 for FT3 and FT4, respectively; *p* <0.001), which was consistent with previous observations [2].

**Table 4:** The response relationship between thyroid function and the serum TC level

**Variable**	**Coefficients**	**Significant frequencies**	**P value**
TSH	0.015580712	89	<0.001
FT3	-0.001921199	56	<0.001
FT4	-0.011725050	97	<0.001

The data are presented as the coefficients and significant frequencies. *P*-values less than 0.05 were considered statistically significant.

## Discussion

The present study reported the relationship between TSH levels and the lipid status after adjustments for the thyroid hormones and/or other potentially confounding factors in patients with CHD. The principal finding is that the prevalence of hypercholesterolemia increased as the serum TSH level increased and TSH *per se* was positive correlated with TC. Although the TSH-induced increase in TC is weak, it may be physiologically relevant and clinically significant. Thyroid function has an important role in the risk stratification of these patients with suspected CHD and should be routinely tested in the patients at risk of CHD. Maintaining the serum TSH levels in an appropriate range will achieve homeostasis of the lipid levels and slow the progression of atherosclerosis in CHD patients.

To ensure our study to have considerable strength, we used more stringent exclusion criteria and the impact of potential confounders was minimized. Firstly, we ruled out the individuals with euthyroid sick syndrome (ESS) as possible as we can. ESS, also known as low triiodothyronine syndrome, is often seen in starvation, critical illness or patients in intensive care unit. The prevalence of ESS was as higher as 29.2% in the patients with cardiovascular diseases [18]. The most prominent alterations in this condition are low serum T3 and elevated rT3 levels [19]. Individuals with such characteristics were excluded. Secondly, the patients enrolled our study were clinically stable. Acute myocardial infarction and any other acute or serious diseases were not included. Third and most important, individuals using drugs that influence serum lipids or thyroid function were excluded. This may sound weird because nearly all CHD patients should take lipid-lowering drugs known as statin and could benefit of statin therapy. However, a great number of Chinese, especially in the peasants, do not see a doctor and take medicine until they could not endure any longer. Deficient knowledge of disease, poor economic conditions and/or no medical insurance may be the main reason.

Studies on the prevalence and distribution of thyroid dysfunction have primarily been carried out in the general population and have rarely been investigated in CHD patients. In 2007, Iervasi *et al.* evaluated 3121 cardiac patients and found that the prevalence rates of subclinical hypothyroidism, subclinical hyperthyroidism and low triiodothyronine syndrome were 6.6%, 3.1% and 29.2%, respectively [18]. In the current study, the prevalence of subclinical hypothyroidism was 10.04%, which is higher than the prevalence reported by Iervasi *et al.* An explanation for this difference could be attributed to the subjects who were studied. The study by Iervasi *et al.* enrolled patients with various types of heart disease, including ischemic (n = 1679) and nonischemic (n = 1442) heart disease. In the present study, however, we selected patients who were diagnosed with coronary heart disease by angiography. In addition, we excluded all of the individuals with euthyroid sick syndrome.

Recently, the associations between TSH and the serum lipid status have become a popular area of research. Interestingly, most of the research regarding the association between TSH and the serum lipid status has been carried out in euthyroidic subjects. Several studies have already found positive correlations between TSH and lipid profiles. The HUNT study, which was performed in Norway, showed linear and significant increases in the serum TC, LDL and TG levels with a TSH level that increased within the reference range [20]. Similar results were also obtained in euthyroidic populations of Korean [21], Latin American [22] and Spanish [23] individuals. However, the relationship between SCH, dyslipidemia and cardiovascular disease is still a topic of debate [24]. In an Austrian study of 1055 patients with SCH and 4856 participants with normal thyroid function, there was no significant difference in the cholesterol levels between the two groups [25]. However, in these studies, the estimates were only adjusted for the traditional serum lipid confounding factors, such as age, gender, BMI and smoking status. It is well known that hypothyroidism is one of the main causes of secondary dyslipidemia. Significant correlations between the levels of TC and both FT3 and FT4 have been observed in patients with thyroid dysfunction [2,3]. An inverse relationship between serum T4 levels and cholesterol was confirmed by almost all of the studies [26]. In addition, the present study found significant negative correlations between the thyroid hormone levels and TC (r = −0.122, *p* = 0.007 for FT3; and r = −0.197, *p* < 0.001 for FT4 to LogTC after adjusting for sex, age, smoking status and FPG). Thus, the thyroid hormones should be considered as important confounding factors when considering the relationship between TSH and lipid parameters. It is essential to evaluate the effects of TSH on lipid profiles independent of the thyroid hormone levels. If corrections are not applied for the important variables that can affect lipid levels, then the association between TSH and the lipid profile is marginal. Even after adjusting for the thyroid hormones in the present study, the significant positive correlation between TSH and LogTC (r = 0.095, *p* = 0.036) persisted. Furthermore, TSH can increase the serum TC level, but the extent of this effect is unknown. Regrettably, few studies have addressed the effects of TSH on TC. We demonstrated that each 1 mIU/L increase in the TSH level tended to elevate the TC level by 0.015580712 mmol/L. A thyroid hormone-independent effect of TSH on the lipid profiles was not found in the previous studies. These innovative and original results clearly indicate an independent relationship between TSH and lipids.

Progress by our laboratory on the function of TSH has provided sensible explanations for our results. Studies have shown that TSH receptors are expressed in a variety of extrathyroidal tissues, including the liver, ovary, testis, skin, kidney, immune system, white and brown adipose tissues, orbital preadipocyte fibroblasts and bone [27]. In addition, a large body of evidence has emerged to describe the functional roles of TSH receptors in these various tissues. Our previous studies have demonstrated that liver cells express TSH receptor protein [28] and that TSH upregulates the expression of HMGCR (the rate-limiting enzyme in cholesterol synthesis) by acting on TSH receptors in the hepatocyte membranes [15]. TSH thereby promotes cholesterol synthesis in the liver and elevates cholesterol levels both *in vivo* and *in vitro*. Furthermore, studies have shown that TSH acts directly on preadipocyte differentiation and adipogenesis [29], stimulates lipolysis in cultured adipocytes and elevates serum free fatty acid levels *in vivo*[30] and affects leptin [31]. However, the direct effects of TSH on lipids require further investigation.

Although the TSH-induced increase in TC is weak, it may have significant clinical implications. One should not overlook the importance of thyroid function which should always be assessed in the patients at risk of CHD, to exclude not only thyroid dysfunction, but also secondary dyslipidemia. The serum TSH levels of these patients with suspected CHD can serve as important biomarkers by which to perform the risk stratification. Furthermore, an increasing number of studies have proved relatively low serum TSH level in reference range is beneficial for CHD patients [6]. Particularly, compared to measurement of other biomarkers, such as C-reactive protein and homocysteine, measurement of TSH is simple and can be performed in most hospitals.

Although the present study identified the direct effects of TSH on lipid levels after correcting for the thyroid hormones in CHD patients, the present study has several limitations. First, because we used a cross-sectional design, the causality between TSH and TC levels cannot be fully established (i.e., we can only suggest an association). A well-designed prospective research study will be necessary to address the relationship between TSH and TC. However, our previous studies [15,28] may provide, to a certain extent, explanations for the positive relationship between TSH and TC. Second, variations in thyroid function due to the influence of an acute setting or heart disease were not able to avoid completely. Thus, some individuals with transiently abnormal TSH levels may have been classified as having a thyroid dysfunction. However, the practice of measuring TSH levels twice and applying more stringent exclusion criteria made the impact of an acute setting or heart disease minimized. In addition, most of studies on thyroid function that have been published have adopted measuring TSH levels at one time point [6,7,10]. Third, the power of the present study could have been stronger with a larger number of study subjects.

## Conclusions

In conclusion, we found that serum TSH levels are positively and linearly associated with serum TC levels after adjustments for the thyroid hormones in CHD patients. In addition, we found that TSH alone can increase the TC level in CHD patients independent of the thyroid hormones. Furthermore, the prevalence of hypercholesterolemia increased with an increasing serum TSH level. Each 1 mIU/L increase in the TSH level was estimated to elevate the TC level by 0.015580712 mmol/L. Although this is a very small effect, it is biologically and clinically significant. In accordance with our previous report that TSH can increase cholesterol synthesis by upregulating HMGCR independent of the thyroid hormones, the present finding that TSH elevations increased the TC level showed a direct clinical effect of TSH on the TC level. Because there is a high prevalence of thyroid dysfunction in CHD patients, it is necessary to routinely test the thyroid function of CHD patients. Maintaining the serum TSH levels in an appropriate range will achieve a homeostasis of the lipid levels and slow the progression of atherosclerosis in CHD patients.

## Abbreviations

SCH, Subclinical hypothyroidism; TSH, Thyroid stimulating hormone; T4, Thyroxine; CHD, Coronary heart disease; BMI, Body mass index; FT4, Free thyroxine; HMGCR, Hepatic 3-hydroxy-3-methyl-glutaryl coenzyme A reductase; T3, Triiodothyronine; FT3, Free triiodothyronine; rT3, Reverse T3; FPG, Free plasma glucose; TC, Total cholesterol; TG, Triglycerides; LDL, Low-density lipoprotein cholesterol; HDL, High-density lipoprotein cholesterol; UA, Uric acid.

## Competing interests

The authors declare that they have no competing interests.

## Authors’ contributions

The work presented here was carried out in collaboration between all authors. Ling Gao and Jiajun Zhao defined the research theme. Chao Xu analyzed the data, interpreted the results and wrote the paper. Xiaomei Yang designed methods, collected data and contributed equally to this study. Wenhui Liu performed the statistical analysis. Haitao Yuan co-worked on associated data collection; Chunxiao Yu participated in the design of the study. All authors have contributed to, read and approved the final manuscript.
